# Human-to-Human Rabies Transmission via Solid Organ Transplantation from a Donor with Undiagnosed Rabies — United States, October 2024–February 2025

**DOI:** 10.15585/mmwr.mm7439a1

**Published:** 2025-12-04

**Authors:** Rebecca Earnest, Kris K. Carter, Sara F. Margrey, Vaughn V. Wicker, Rebecca Betz, Rebecca Reik, Eli Shiltz, Basmah Khalil, Brandon Palinski, Barbara Jordan, Daniel Dodson, Erin Epson, Curtis L. Fritz, Juliet Stoltey, Alison Sikola, Ricardo Garcia, Monika Roy, Pallavi Annambhotla, Sridhar V. Basavaraju, Sarah C. Bonaparte, Shama Cash-Goldwasser, Ian Kracalik, David W. McCormick, Faisal S. Minhaj, Lillian A. Orciari, Panayampalli S. Satheshkumar, Pamela Yager, David A. Crum, Nathan Koffarnus, Monica Beddo, Molly Baker, Erin C. Phipps, Hanna Oltean, Hannah Schnitzler, Crystal M. Gigante, Ryan M. Wallace, Mary Grace Stobierski, Christine Hahn

**Affiliations:** ^1^Division of High-Consequence Pathogens and Pathology, National Center for Emerging and Zoonotic Infectious Diseases, CDC; ^2^Epidemic Intelligence Service, CDC; ^3^Idaho Department of Health and Welfare; ^4^Division of State and Local Readiness, Office of Readiness and Response, CDC; ^5^Ohio Department of Health; ^6^Idaho Panhandle Health District, Hayden, Idaho; ^7^Michigan Department of Health & Human Services; ^8^The University of Toledo, Toledo, Ohio; ^9^Toledo-Lucas County Health Department, Toledo, Ohio; ^10^California Department of Public Health, Richmond and Sacramento, California; ^11^County of Santa Clara Public Health Department, San Jose, California; ^12^Division of Healthcare Quality Promotion, National Center for Emerging and Zoonotic Infectious Diseases, CDC; ^13^Maryland Department of Health, Baltimore, Maryland; ^14^Missouri Department of Health and Senior Services, Springfield, Missouri; ^15^New Mexico Department of Health; ^16^Washington State Department of Health, Tumwater, Washington.

**Summary**

**What is already known about this topic?**

Although rabies virus is typically transmitted through bites or scratches from an infected animal, human-to-human transmission has occurred through organ and tissue transplantation. Rabies is almost always fatal without postexposure prophylaxis (PEP).

**What is added by this report?**

In February 2025, CDC confirmed a fatal rabies case in a patient who had received a transplanted kidney from a deceased donor with undiagnosed rabies. Three cornea recipients from the same donor underwent graft removal, received PEP, and remained asymptomatic. Risk assessments for 357 of 370 (96%) possible contacts were completed, 46 (13%) of whom were recommended to receive PEP.

**What are the implications for public health practice?**

This was the fourth transplant-transmitted rabies event in the United States since 1978. Early public health consultation might help prevent donation of rabies-infected organs and tissue. PEP assessment should be considered when potential rabies exposures are identified in donors.

## Abstract

Although rabies virus is typically transmitted through mammalian animal bites or scratches, human-to-human transmission has occurred through organ and tissue transplantation. From 1978 to 2013, three transplant-transmitted rabies events in the United States affected nine tissue or organ recipients. Rabies is almost always fatal without timely receipt of postexposure prophylaxis (PEP). In January 2025, clinicians in Ohio notified the Ohio Department of Health and CDC of a suspected case of rabies in a kidney transplant recipient who died 51 days after receiving the transplant. CDC confirmed the recipient’s rabies diagnosis. Investigation revealed that the deceased donor had been scratched by a skunk approximately 6 weeks before death. No other organs from that donor were transplanted; however, three persons received cornea tissue grafts. While investigation of the donor’s rabies status was ongoing, the cornea recipients underwent precautionary graft removal and received PEP. None developed signs or symptoms compatible with rabies. CDC detected rabies virus RNA in an archived sample of the donor’s kidney, confirming organ-derived transmission. Investigation identified 370 persons with possible exposures to the donor or kidney recipient; 357 (96%) completed risk assessments. Among those who completed risk assessments, 46 (13%) were recommended to receive PEP. Early consultation with public health officials might prevent rabies-infected organ and tissue donation or lead to prompt PEP for transplant recipients. The risk for rabies should be considered among donors who have received rabies-susceptible animal bites or scratches within the previous year, particularly those donors with acute encephalopathy.

## Investigation and Results

### Notification of Rabies Case

On January 27, 2025, CDC was notified of possible rabies virus infection in a kidney transplant recipient (Figure). After CDC confirmed diagnosis, jurisdictional health departments and CDC conducted an investigation to determine the source of the infection and to identify other persons at risk. This activity was reviewed by CDC, deemed not research, and was conducted consistent with applicable federal law and CDC policy.*

**FIGURE:**
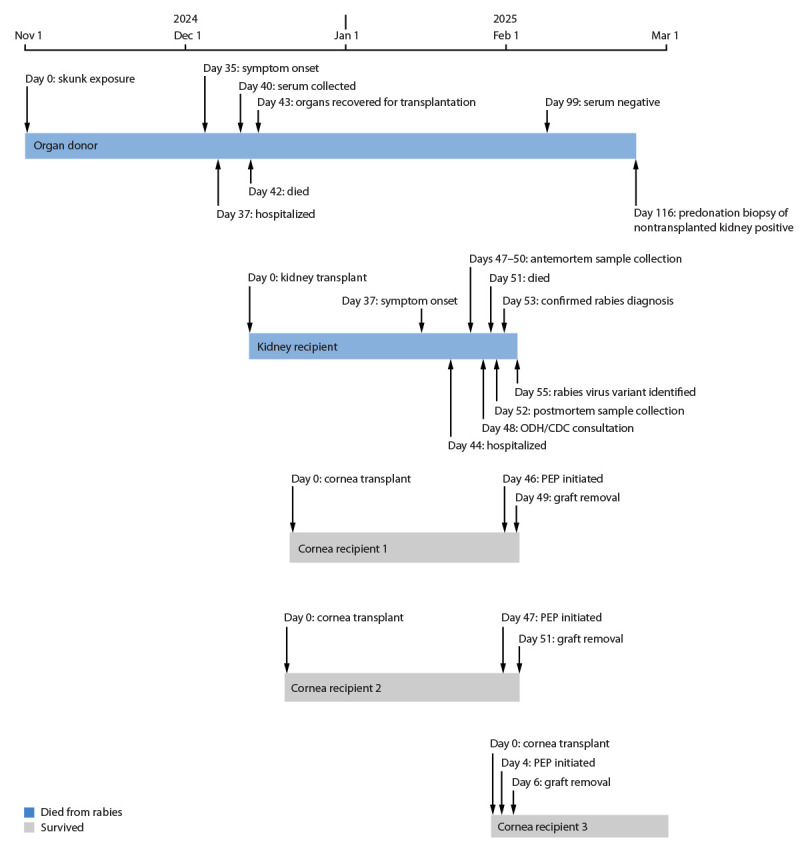
Clinical course, diagnostic testing, and investigation timeline for a transplant-associated rabies case* — United States, November 2024–February 2025 **Abbreviations:** ODH = Ohio Department of Health; PEP = postexposure prophylaxis. * Antemortem samples from the kidney recipient included cerebrospinal fluid (collected on day 47), skin and serum samples (day 49), and saliva (days 49–50).

### Kidney Recipient

In December 2024, an adult male Michigan resident received a left kidney transplant from an Idaho donor at an Ohio hospital. Approximately 5 weeks later, the recipient experienced tremors, lower extremity weakness, confusion, and urinary incontinence. Seven days after symptom onset, he was hospitalized with fever, hydrophobia, dysphagia, and autonomic instability. On hospital day 2, he required invasive mechanical ventilation. On hospital day 4, clinicians consulted the Ohio Department of Health and CDC because the recipient’s signs and symptoms were compatible with rabies. The Donor Risk Assessment Interview (DRAI) questionnaire reported that the donor had received a skunk scratch. The kidney recipient’s saliva, nuchal skin biopsy, serum, and cerebrospinal fluid samples, collected on hospital days 3–6, were sent to CDC for rabies testing. On hospital day 7, the recipient died; postmortem brain tissue samples were sent to CDC. Rabies virus RNA was detected in saliva, nuchal skin, and brain tissue samples. Rabies virus immunoglobulin (Ig) M, IgG, and neutralizing antibodies were detected in serum. Viral characterization was consistent with the silver-haired bat (*Lasionycteris noctivagans*) rabies virus variant. Michigan public health officials interviewed the kidney recipient’s family; no animal exposures were reported.

### Idaho Organ Donor

After rabies was suspected, the Idaho Department of Health and Welfare, the local public health district, and CDC investigated the Idaho donor as the possible infection source. Interviews with the family added details not included in the DRAI questionnaire. In late October 2024, a skunk approached the donor as he held a kitten in an outbuilding on his rural property. During an encounter that rendered the skunk unconscious, the donor sustained a shin scratch that bled, but he did not think he had been bitten. According to the family, the donor attributed the skunk’s behavior to predatory aggression toward the kitten.

A member of the donor’s household reported that approximately 5 weeks later, in early December, the donor was confused, had difficulty swallowing and walking, experienced hallucinations, and had a stiff neck. Two days after symptom onset, he was found unresponsive at home after presumed cardiac arrest. He was resuscitated and hospitalized but never regained consciousness. He was declared brain dead and removed from life support on hospital day 5. Left kidney, heart, lungs, and both corneas were recovered.

After rabies was suspected in the kidney recipient, stored serum that had been collected from the donor on his third hospital day was tested and determined to be negative for rabies virus antibody. A multiweek laboratory traceback investigation identified right and left kidney biopsy samples. CDC detected rabies virus RNA consistent with a silver-haired bat rabies virus variant in a biopsy sample of the right kidney, suggesting organ-derived transmission. The left kidney biopsy sample had insufficient tissue for testing.

### Donor Corneal Tissues and Tissue Recipients

Four ocular grafts were prepared from recovered corneas. Three patients, one each from California, Idaho, and New Mexico, received grafts in December 2024 and January 2025. While investigation of the donor’s rabies status was ongoing, the cornea recipients underwent precautionary graft removal and received PEP. They remained asymptomatic. A planned transplantation of the fourth corneal graft to a Missouri patient was cancelled. CDC detected rabies virus RNA consistent with a silver-haired bat rabies virus variant in one previously implanted corneal graft.

### Donor Heart and Lungs

The heart and lungs of the donor were not transplanted but were used in training procedures at a Maryland medical research facility. By the time of the public health investigation, no specimens were available for testing.

## Public Health Response

### Rabies Risk Assessments and PEP Recommendations

Rabies virus detection in the kidney recipient prompted investigations to identify persons who were exposed to either the presumed rabid skunk, the donor, or the kidney recipient during their respective infectious periods. Rabies virus can be transmitted via direct contact of saliva, tears, or innervated tissues (e.g., organs and other body tissues) from infected humans or animals with broken skin or mucous membranes. Infectious periods were defined as 14 days before symptom onset until death and decontamination (*1*). Public health officials, agency medical directors, and occupational health staff members conducted exposure risk assessments to determine which contacts should consult their provider to receive the appropriate PEP course (Table). Data on the percentage of contacts recommended PEP who received it were unavailable.

**TABLE:** Close contacts, rabies exposure risk assessments, and postexposure prophylaxis recommendations, by exposure group — United States, November 2024–February 2025

Exposure group	No. of possible contacts*	No. (%)	
		Received exposure risk assessment^†^	Recommended to receive PEP^§^
**Kidney recipient**			
Community member	14	14 (100)	6 (43)
Health care worker^¶^	269	256 (95)	16 (6)
Cornea recipient	—	—	—
**Total**	**283**	**270 (95)**	**22 (8)**
**Organ donor**			
Community member	4	4 (100)	4 (100)
Health care worker^¶^	80	80 (100)	17 (21)
Cornea recipient	3	3 (100)	3 (100)
**Total**	**87**	**87 (100)**	**24 (28)**

**Abbreviation**: PEP = postexposure prophylaxis.

* Kidney transplant recipient excluded from possible contacts of organ donor.

^†^ Percent assessed = number assessed / number of possible contacts.

^§^ Percent recommended PEP = number recommended PEP / number assessed. Data on the percentage of close contacts recommended to receive PEP who did receive it were unavailable.

^¶^ Includes persons handling donor or kidney recipient organs or tissues in health care or research settings.

**Persons exposed to donor and skunk.** No other persons or animals were exposed to the presumed rabid skunk. Four community contacts were identified and completed risk assessments; all were recommended to receive PEP. An additional 80 health care worker contacts were identified and completed risk assessments; 17 (21%) were recommended to receive PEP. The three cornea recipients received PEP, consisting of human rabies immunoglobulin and 4 rabies vaccine doses, and antibody testing to confirm adequate vaccination responses.

**Persons exposed to kidney recipient.** Fourteen community contacts were identified and completed risk assessments; six were recommended to receive PEP. An additional 269 health care worker contacts were identified, 256 (95%) of whom completed risk assessments; 16 (6%) were recommended to receive PEP.

## Discussion

A diagnosis of rabies in a kidney transplant recipient with no recognized animal exposure resulted in a multistate public health investigation to ascertain whether the kidney donor had undiagnosed rabies, identify other donor organs and tissues, and identify rabies-exposed persons. Laboratory testing and viral characterization identified rabies virus infection in the donor, confirming organ-derived transmission.

Rabies risk varies by species, geography, and exposure circumstances (*2*). Rabies virus is maintained by specific animal reservoir species in the continental United States (i.e., bats, raccoons, skunks, and foxes); however, any mammal can be infected, and approximately 800 cross-species infections were reported in 2022 (*3*). Although skunks are not a rabies virus reservoir in Idaho, bat-maintained rabies virus variants, including the silver-haired bat variant, are enzootic in the state. Investigation suggested a likely three-step transmission chain in which a rabid silver-haired bat infected a skunk, which infected the donor and led to infection of the kidney recipient. Contact tracing, graft removal, and PEP administration mitigated possible rabies risk to cornea recipients. Risk assessments led to PEP recommendations for 46 of 357 (13%) contacts.

This was the fourth reported transplant-transmitted rabies event in the United States since 1978 (*4*–*6*); however, the risk for any transplant-transmitted infection, including rabies, is low (*7*). Among 13 recipients involved in the four transplant-transmitted rabies events, seven recipients who did not receive PEP died, and all six who received PEP survived (*8*). Limited data suggest that, when appropriately administered before symptom onset, PEP can prevent rabies disease among transplant recipients from infected donors (*8*). Worldwide, two PEP failures have been reported among 21 organ and tissue recipients (*9*,*10*). One recipient initiated but declined to complete the PEP series (*9*); the reason for failure in the other recipient is unclear (*10*). In addition to receiving PEP, all three cornea graft recipients underwent precautionary graft removal. Receipt of any organs or tissues from a rabid donor is a high-risk exposure; however, the proximity of corneal and optic nerves to the central nervous system raises additional concerns for shortened incubation periods. In these cases, cornea graft removal could further reduce the risk. The relative ease of cornea graft removal, compared with life-sustaining organs or other tissue types, could also influence clinical decision-making.

In the United States, potential donors’ family members often provide information about a donor’s infectious disease risk factors, including animal exposures. Rabies is excluded from routine donor pathogen testing because of its rarity in humans in the United States and the complexity of diagnostic testing. In this case, hospital staff members who treated the donor were initially unaware of the skunk scratch and attributed his preadmission signs and symptoms to chronic comorbidities. After the kidney recipient’s symptom onset, the donor’s DRAI-documented skunk exposure contributed to suspicion for rabies. The resulting response identified the donor as the infection source and mitigated the possible risk to others.

### Implications for Public Health Practice

CDC, the Health Resources and Services Administration, and partners are reviewing the occurrence of reported exposures to animals among donors to identify interventions to further reduce transplant-associated rabies risk. No standard guidance currently exists for addressing reported donor animal exposures by transplant teams. If a potential donor, particularly one with acute encephalopathy, had a bite or scratch from a rabies-susceptible animal during the preceding year, transplant teams should consider consulting public health officials to determine rabies risk.

A public health consultation can occur before or shortly after transplantation. The rabies incubation period is often weeks to months, and the Advisory Committee on Immunization Practices in 2008 determined that PEP decisions are a medical urgency, not an emergency (*2*). If an organ or tissue has been transplanted from a donor who is subsequently suspected to have had rabies, a risk assessment could save lives by accelerating diagnostic testing, possible explantation when deemed clinically appropriate, and PEP administration to recipients and other contacts.

## Footnotes

* 45 C.F.R. part 46, 21 C.F.R. part 56; 42 U.S.C. Sect. 241(d); 5 U.S.C. Sect. 552a; 44 U.S.C. Sect. 3501 et seq.
